# Osteomielitis craneofacial en un paciente con compromiso sistémico: reporte de un caso

**DOI:** 10.21142/2523-2754-1203-2024-213

**Published:** 2024-09-17

**Authors:** Xiomara Zilena Serpa-Romero, Gustavo Julio Guzmán-Maciel, Raúl Montes-García, Camilo A. Romo-Pérez

**Affiliations:** 1 Facultad de Odontología, Universidad del Magdalena. Santa Marta, Colombia. xiomaraserpa@gmail.com Universidad del Magdalena Facultad de Odontología Universidad del Magdalena Santa Marta Colombia xiomaraserpa@gmail.com; 2 Departamento Cirugía Maxilofacial. Clínica Mar Caribe, Santa Marta, Colombia. gguzman@unimagdalena.edu.co Departamento Cirugía Maxilofacial Clínica Mar Caribe Santa Marta Colombia gguzman@unimagdalena.edu.co; 3 Universidad del Magdalena. Santa Marta, Colombia. raulmontesag@unimagdalena.edu.co Universidad del Magdalena Universidad del Magdalena Santa Marta Colombia raulmontesag@unimagdalena.edu.co; 4 Fundación Universitaria San Martín. Puerto Colombia, Colombia. camilo.romo@sanmartin.edu.co Universidad San Martín Fundación Universitaria San Martín Puerto Colombia Colombia camilo.romo@sanmartin.edu.co

**Keywords:** osteomielitis, osteonecrosis, pos-COVID-19, absceso cerebral, osteomyelitis, osteonecrosis, post-COVID-19, brain abscess

## Abstract

Este informe presenta el caso de un paciente masculino de 61 años con diabetes, hipertensión y antecedentes de COVID-19 grave, con osteomielitis del seno frontal y maxilar superior. El paciente refirió dolor intenso en la zona facial y maxilar durante dos meses. Las imágenes de tomografía computarizada mostraron un engrosamiento mucoperióstico del seno maxilar y la resonancia magnética mostró múltiples lesiones líticas a nivel del seno frontal, así como abscesos cerebrales en la región frontal izquierda que se correlacionaron con los cultivos microbiológicos. En el examen clínico intraoral se observaron segmentos con aspecto necrótico del maxilar superior, fístula orosinusal y múltiples focos infecciosos dentales. El paciente fue tratado con antibióticos, analgésicos, antiinflamatorios, insulina e inhibidores de la bomba de protones. Se realizó trepanación del seno frontal, drenaje del absceso y secuestrotomía en el maxilar superior, y se solicitaron sesiones con cámara hiperbárica.

## SUMMARY

This report presents the case of a 61-year-old male patient with diabetes, hypertension, and a history of severe COVID-19 with osteomyelitis of the frontal sinus and upper jaw. The patient reported intense pain in the facial and maxillary area for two months. The computed tomography images showed mucoperiosteal thickening of the maxillary sinus and the magnetic resonance imaging showed multiple lytic lesions at the frontal sinus level, as well as brain abscesses in the left frontal region that were correlated with the microbiological cultures. In the intraoral clinical examination, segments with a necrotic appearance of the upper jaw, orosinusal fistula and multiple dental infectious foci were observed. The patient was treated with antibiotics, analgesics, anti-inflammatories, insulin, and proton pump inhibitor. Trephination of the frontal sinus, drainage of the abscess and sequestrectomy in the maxilla were performed and sessions with a hyperbaric chamber were ordered.

## INTRODUCCIÓN

La pandemia de COVID-19 que apareció a principios de 2020 y se extendió hasta mediados de 2022 se ha relacionado con múltiples complicaciones de los sistemas respiratorio, cardiovascular y renal ^1^. Asimismo, se notificaron varias complicaciones en el área maxilofacial debido a estas infecciones respiratorias, entre las que destacan la osteomielitis, la osteonecrosis de la mandíbula, la trombosis de los senos cavernosos y las complicaciones neurológicas ^2^^,^^3^.

La osteomielitis es una infección del hueso que puede ser aguda o crónica, caracterizada por la inflamación del tejido óseo y la médula ósea. Esta condición puede resultar de infecciones bacterianas, fúngicas o, incluso, por la extensión de infecciones de tejidos adyacentes. En el contexto de la osteomielitis maxilar, esta es menos común que en la mandíbula debido a la mayor vascularización del maxilar, lo que lo hace menos susceptible a infecciones. La osteomielitis se manifiesta a menudo con síntomas como dolor localizado, hinchazón y, en algunos casos, drenaje de pus. El diagnóstico se realiza mediante estudios de imagen, como radiografías, tomografías computarizadas o resonancias magnéticas, y el tratamiento generalmente incluye antibióticos y, en casos severos, cirugía para desbridar el tejido infectado ^4^.

Los informes anteriores de personas con una historia clínica posterior a la COVID-19 indican varias posibles vías fisiopatogénicas para el desarrollo de complicaciones en el área ósea maxilofacial. En el caso de la osteomielitis por osteonecrosis, la teoría más apoyada afirma que las coagulopatías y el desarrollo de microtrombos producidos en esta afección alteran la microcirculación, lo que da lugar a un estado isquémico local que posiblemente sea la causa de la necrosis ósea en los maxilares ^3^.

Además, patologías sistémicas crónicas como la diabetes mellitus constituyen un factor de riesgo para el desarrollo de complicaciones infecciosas y la disminución de la capacidad de cicatrización ^5^. Por otra parte, algunos informes sugieren un mayor riesgo de osteomielitis-osteonecrosis debido a la susceptibilidad a las infecciones causadas por el tratamiento antiinflamatorio con corticosteroides utilizado principalmente en las fases graves de la COVID-19, y debido a una marcada disminución de las células T CD4+ y CD8+ que puede llevar a los pacientes a un estado de inmunosupresión incluso en la fase posterior a esta enfermedad ^3^^,^^6^^,^^7^.

Resulta fundamental que la comunidad médica esté atenta a las complicaciones maxilofaciales que pueden surgir en la población que ha padecido COVID-19. La identificación temprana de síntomas y la evaluación adecuada de los pacientes son esenciales para prevenir el desarrollo de condiciones severas como la osteomielitis. Además, se requiere una investigación más profunda para comprender mejor las relaciones entre esta patología y las complicaciones maxilofaciales, lo que permitirá establecer protocolos de tratamiento más efectivos y estrategias de prevención. La educación de los profesionales de la salud sobre estas posibles complicaciones también es crucial para garantizar que los pacientes reciban una atención adecuada y oportuna.

### Consideraciones metodológicas y éticas

Se presenta un caso clínico basado en las recomendaciones de la Case Report Statement and Checklist (CARE) ^8^, disponible en http://www.equator-network.org/. Este estudio siguió siempre los lineamientos de los principios de investigación en seres humanos, la Declaración de Helsinki de 1975, la resolución 8430 de 1993 del Ministerio de Salud de Colombia y Ley Estatutaria 1581 de 2012 para la protección de datos personales. La autorización para el acceso a la historia clínica de la paciente fue otorgada por el Comité de Ética de la Clínica Mar Caribe, en Santa Marta, Magdalena (Colombia). Adicionalmente, el paciente firmó un consentimiento informado.

### Reporte de caso 

Paciente masculino de 61 años que acudió al servicio de urgencias de una institución hospitalaria de alta complejidad en Santa Marta, Colombia, con la consulta “me duele mucho la cara”, el cual fue remitido desde el servicio de cirugía oral y maxilofacial. con diagnóstico inicial de sinusitis etmoidal aguda. La paciente refería antecedentes sistémicos de hipertensión arterial bajo prescripción con metoprolol 100 mg dos veces al día, diabetes mellitus tipo II sin control farmacológico manejada por compensación alimentaria y antecedente de COVID-19 severa hace dos meses, que requirió manejo en la unidad de cuidados intensivos mediante terapia antiinflamatoria con dos dosis de tocilizumab IV.

El paciente presentaba dolor desde hacía dos semanas y empeoró dos días antes del ingreso en la consulta. El dolor fue descrito como pulsátil localizado, de gran intensidad en la región frontal del cráneo y etmoidal derecha, asociado a drenaje nasal tras rinoscopia realizada antes de la consulta con informe de sinusitis etmoidal izquierda (programada para etmoidectomía anterior y posterior) y celulitis periorbitaria.

Al ingreso hospitalario, se solicitaron pruebas paraclínicas que revelaron leucocitos con 15,100/mm^3^ y glucemia de 290mg/dl. Se instauró tratamiento antimicrobiano, antiinflamatorio y analgésico con ampicilina/sulbactam 3 g IV cada 6 h, dexametasona 8 mg IV cada 8 h, dipirona diluida 3 mg IV cada 8 h, y se complementó el tratamiento farmacológico con insulina glargina 15 u pm y omeprazol 40 mg IV cada 24 horas.

**Figura 1:**
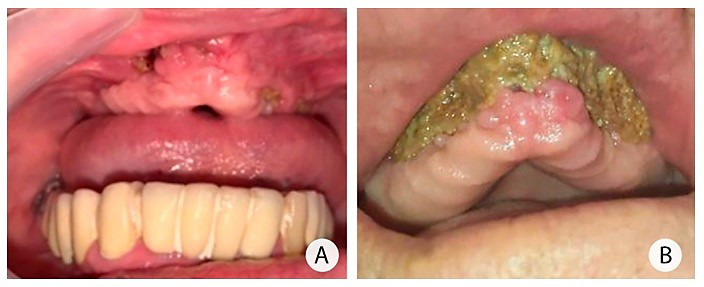
Fotografías intraorales. A) Secuestro óseo maxilar bilateral, el cual se manifiesta mediante múltiples trayectos fistulosos. Estos se presentan de forma simétrica y comprometen una extensa porción del tejido óseo maxilar. B) La evolución crónica y la progresión de la osteomielitis maxilar han desencadenado la formación de una lesión de secuestro óseo de gran magnitud. Esta lesión abarca más del 50% de la estructura ósea del maxilar.

El examen oral realizado por el departamento de cirugía maxilofacial reveló la presencia de segmentos con aspecto necrótico del maxilar superior, restauraciones metal-porcelana inadaptadas, múltiples focos de infecciones en órganos dentarios y la presencia de una comunicación oro-antral derecha (Figura 1a). El paciente fue tratado con antibioticoterapia parenteral (cefepime, meropenem 2 g IV cada 8 h), vancomicina (1,5 g IV cada 12 h), y se utilizó terapia antifúngica parenteral (fluconazol 400 mg IV cada 12 h) (Figuras 1a y 1b).

**Figura 2:**
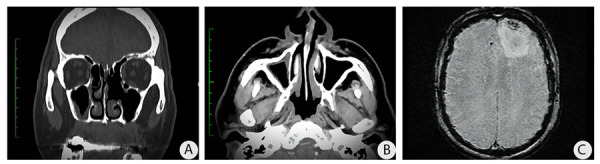
Imágenes radiológicas iniciales. A) Engrosamiento de la pared basal del seno maxilar derecho con disminución del segmento óseo relacionado con el maxilar superior, comunicación oro sinusal en la pared basal del seno maxilar izquierdo. Tomografía axial computarizada. B) Se evidencia presencia de líquido acumulado en el seno maxilar izquierdo, con disminución de la pared medial. C) Abscesos cerebrales en región frontal izquierda en fase capsular tardía, con discreto edema cerebral vasogénico periférico. Realce paquimeníngeo y leptomeníngeo generalizado.

La exploración física reveló edema, eritema y nodulación en el párpado derecho. Las imágenes tomográficas describían cambios en la tabla ósea craneal, lo que sugiere descartar un mieloma múltiple. En la tabla ósea craneal se observaron múltiples lesiones líticas de aspecto salino. También se observó engrosamiento mucoperióstico del seno maxilar derecho (Figuras 1a y 1b). Se realizó una resonancia magnética (RM) (figura 2c) donde se observaron abscesos cerebrales en la región frontal izquierda (corte RM: 37/60) en fase capsular tardía, con discreto edema cerebral vasogénico periférico, correlacionando con los resultados de los análisis microbiológicos.

### Procedimiento quirúrgico

Bajo anestesia general, previa asepsia, antisepsia, colocación de campos quirúrgicos y con el paciente en decúbito supino con la cabeza a cero grados, siguiendo el pliegue de expresión. Se realizó una incisión frontal transversa, se disecó por planos hasta el hueso y se realizó el procedimiento. Se siguió con motor quirúrgico y fresa de 5 mm, se realizó trepanación de la pared anterior del seno frontal donde se observó mucosa engrosada con material purulento, el cual fue aspirado, cureteado e irrigado con abundante solución salina y peróxido de hidrógeno. Finalmente, se colocó una esponja estéril absorbible y se reposicionó el colgajo de galea pediculado. Posteriormente, se realizó una cranealizacion del seno frontal mediante trepanación con fresa y aspiración del absceso sin complicaciones mediante catéter n.º 14. El colgajo de galea se suturó con vicryl del 2/0 y la piel, con suturas simples de nylon del 4/0. En el área de cirugía maxilofacial se realizó extirpación quirúrgica del secuestro óseo, drenaje, legrado y posterior lavado de la zona con aspiración de secreciones.

Se enviaron para estudio muestras de secreción sinusal, abscesos cerebrales frontales y mucosa sinusal, informándose como etiquetado cultivo n.º 1 de secreción de absceso intracerebral: negativo para gérmenes aerobios; cultivo n.º 2 de seno frontal (muestra de jeringa líquida): germen aislado: *Klebsiella pneumoniae*, ssp *pneumoniae* y cultivo de seno frontal n.º 3: (muestra de tejido) positivo, germen aislado: *Enterobacter cloacae* complex.

Los diagnósticos definitivos fueron meningoencefalitis bacteriana, absceso cerebral frontal y pansinusitis con osteomielitis frontal y maxilar con foco infeccioso primario: osteonecrosis-osteomielitis maxilar derivada de la comunicación oro-antral tras una extracción dental por COVID-19; como factor precipitante del proceso infeccioso, la aplicación del corticoide tocilizumab por neumonía grave debido al COVID-19. Del mismo modo, se estableció la comunicación oro-antral derecha como factor condicionante de la evolución desfavorable de la paciente, que continuó activa hasta que la paciente fue tratada.

**Figura 3:**
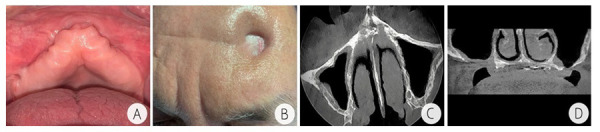
Fotografías y tomografía posquirúrgicas. A) Mucosa alveolar del maxilar superior con color rosa coral después del proceso de cicatrización. B) Proceso de cicatrización tras la intervención de la fístula en la región frontal izquierda. C) Seno maxilar derecho e izquierdo neumatizado, con ligero engrosamiento de la mucosa sinusal, pérdida de la continuidad de la cortical de la pared anterior, sin aparente perforación de la membrana de Schneider. D) Se observa área hipodensa en proceso de cicatrización, en zona anterior del maxilar superior, espina nasal anterior, trabeculado heterogéneo, laxo-esponjoso, corticales parcialmente continua.

El paciente fue hospitalizado con medicación parenteral y órdenes de pruebas hematológicas seriadas cada 5 días, hemograma completo, BUM, creatinina, ionograma, OCT, TGP, VSG y PCR. Como coadyuvante para mejorar el cuadro clínico, se ordenaron 20 sesiones de cámara hiperbárica de 1,5 ATA (atmósferas absolutas) durante 2 horas/día para oxigenar los tejidos y mejorar su respuesta biológica a la cicatrización. Se programó una consulta de reevaluación a los 5 meses en la que no se evidenciaron secreciones en las zonas operadas. El paciente refirió dolor en la zona maxilofacial tratada con levofloxacino 750 mg vía oral. En general, a partir de las terapias con cámara hiperbárica el mejoramiento fue bastante notorio. En las figuras se aprecia el estado actual del paciente, con una mucosa de color rosa coral, bien humectada, que muestra la salud clínica del paciente (Figura 3a) y la cicatrización de la fístula frontal (Figura 3b). Podemos observar también que, a pesar de la pérdida de cortical, no hay perforación de la membrana de Schneider (Figura 3c), lo cual es algo positivo si lo comparamos con el estado inicial en que llegó el paciente.

Los últimos exámenes de resonancia magnética (RM) del paciente muestran una evolución favorable del proceso osteomielítico. En comparación con las RM previos, se observa una disminución significativa del edema y la infiltración de la médula ósea a nivel del maxilar superior, así como una reducción del área de destrucción ósea y la extensión de la lesión. Además, los hallazgos revelan una mejor definición de los márgenes óseos, con menor compromiso de los senos paranasales y una menor reacción inflamatoria de los tejidos blandos adyacentes. Estos cambios positivos en la imagen por RM respaldan la mejoría clínica del paciente y la eficacia del tratamiento antibiótico y quirúrgico. La evaluación detallada de los estudios de RM ha sido fundamental para monitorear la progresión de la osteomielitis y orientar la toma de decisiones terapéuticas en este caso.

## DISCUSIÓN

Las enfermedades crónicas no transmisibles (ECNT), como la diabetes mellitus, las enfermedades cardiovasculares, las enfermedades respiratorias crónicas y el cáncer, se han identificado como factores de riesgo para desarrollar formas graves de COVID-19 y presentar peores desenlaces clínicos. Esto se debe a que estas condiciones crónicas pueden alterar la respuesta inmune del organismo, lo que aumenta la susceptibilidad a infecciones y complicaciones ^1^.

La pandemia de COVID-19 ha incrementado la incidencia de infecciones oportunistas, incluidas las fúngicas y bacterianas, en pacientes que han padecido la enfermedad. Esto se atribuye a la desregulación del sistema inmunológico causada por la infección por SARS-CoV-2 y al uso de terapias inmunosupresoras, como los corticosteroides, durante su manejo. Estos factores predisponen a los pacientes al desarrollo de osteonecrosis del maxilar, especialmente en aquellos con comorbilidades subyacentes como la diabetes mellitus ^9^. De la misma forma, Huh *et al*. ^10^ señalan que condiciones sistémicas como la diabetes alteran la resistencia del paciente a las infecciones y causan complicaciones en el proceso de control y cicatrización.

Desde la pandemia, se ha notificado un aumento considerable de la incidencia de infecciones fúngicas y bacterianas secundarias a esta infección, ya sea durante la enfermedad o como manifestación posterior. Por lo tanto, la aparición de infecciones orales en los maxilares debe ser una señal de alarma para el paciente y para el personal de salud bucodental, incluso en periodos de remisión de la COVID-19 ^11^.

Con relación a los microorganismos presentes en los casos de osteomielitis y COVID-19, se evidencia que, en su mayoría, son de origen fúngico ^10^^,^^12^, lo que contrasta con el trabajo presentado, en el que se pudo identificar la presencia de microorganismos como *Klebsiella pneumoniae* ssp *pneumoniae* y el complejo *Enterobacter cloacae*, pero concuerda con el trabajo de Khan *et al*. ^13^, que comunicaron 13 casos de osteomielitis maxilar que afectaban principalmente al maxilar superior y en cuyos cultivos bacterianos se identificaron *K*. *pneumoniae* y otros gramnegativos, como *E. coli* y pseudomonas.

La comunicación oro-antral que presentaba el paciente de este caso asociada a su evolución desfavorable coincide también con el informe de Khan *et al*. ^13^, quienes informaron que la comunicación orosinusal como complicación frecuente se presentó en 5 de los 13 casos documentados.

Este relato de caso proporciona informaciones que destacan la importancia de realizar una entrevista minuciosa sobre los antecedentes mórbidos de los pacientes, con énfasis en la necesidad de consultar antecedentes de administración de corticoesteroides en pacientes pos-COVID-19, principalmente en casos moderados y graves, ya que, según Suresh *et al*. ^14^, estos pueden desempeñar un papel importante en comprometer aún más la inmunidad disminuida de los pacientes.

No obstante, también se hace un llamado a los odontólogos, estomatólogos y cirujanos maxilofaciales para que tengan en cuenta que el uso de corticoesteroides, la diabetes, las coagulopatías y las coinfecciones son factores de riesgo de osteomielitis en pacientes pos-COVID-19. Además, procedimientos odontológicos como la extracción de dientes o la presencia de focos infecciosos orales preexistentes también pueden contribuir al desarrollo de esta complicación.

Se insta a los profesionales de la salud bucal a mantener un alto índice de sospecha y realizar una evaluación exhaustiva de estos pacientes, incluso en períodos de remisión de la COVID-19. Asimismo, se recomienda que los casos de osteomielitis maxilar en pacientes que han padecido esta enfermedad sean reportados y compartidos en la literatura científica, con el fin de ampliar el conocimiento sobre esta entidad emergente y fortalecer las estrategias de prevención y manejo. 

Un abordaje multidisciplinario, que involucre a especialistas en odontología, infectología y medicina interna, será clave para abordar de manera integral la problemática de la osteomielitis en este contexto. Solo a través de la colaboración y el reporte de más casos clínicos podremos mejorar la comprensión y el manejo de esta complicación en pacientes recuperados de COVID-19.

## Financiamiento:

Los autores no tienen recursos que declarar ni han recibido ningún tipo de financiación de organizaciones privadas, públicas, nacionales o internacionales ni de otra similar
